# Supplementary material for: CLEC18A interacts with sulfated glycosaminoglycans and controls clear cell renal cell carcinoma progression
Source: FEBS J. 2025 Aug 23;293(3):709–28. doi: 10.1111/febs.70236 (PMC12871919; doi:10.1111/febs.70236)
Supplement: Supplementary file 1 — Fig. S1. Conservation and expression of CLEC18A. Fig. S2. scRNA‐seq clustering of fetal kidney and adult kidney cells as according to Stewart B.J. et al 2019. Fig. S3. CLEC18 CRDs have unique phylogeny and ligands. Fig. S4. CLEC18A‐Fc binds cell lines with varying efficiency. Fig. S5. CLEC18 exclusively promotes survival in ccRCC/KIRC. Fig. S6. CLEC18A expression restricts tumor progression in a murine model of renal carcinoma. [file FEBS-293-709-s001.pdf]

Supplementary Figure 1.

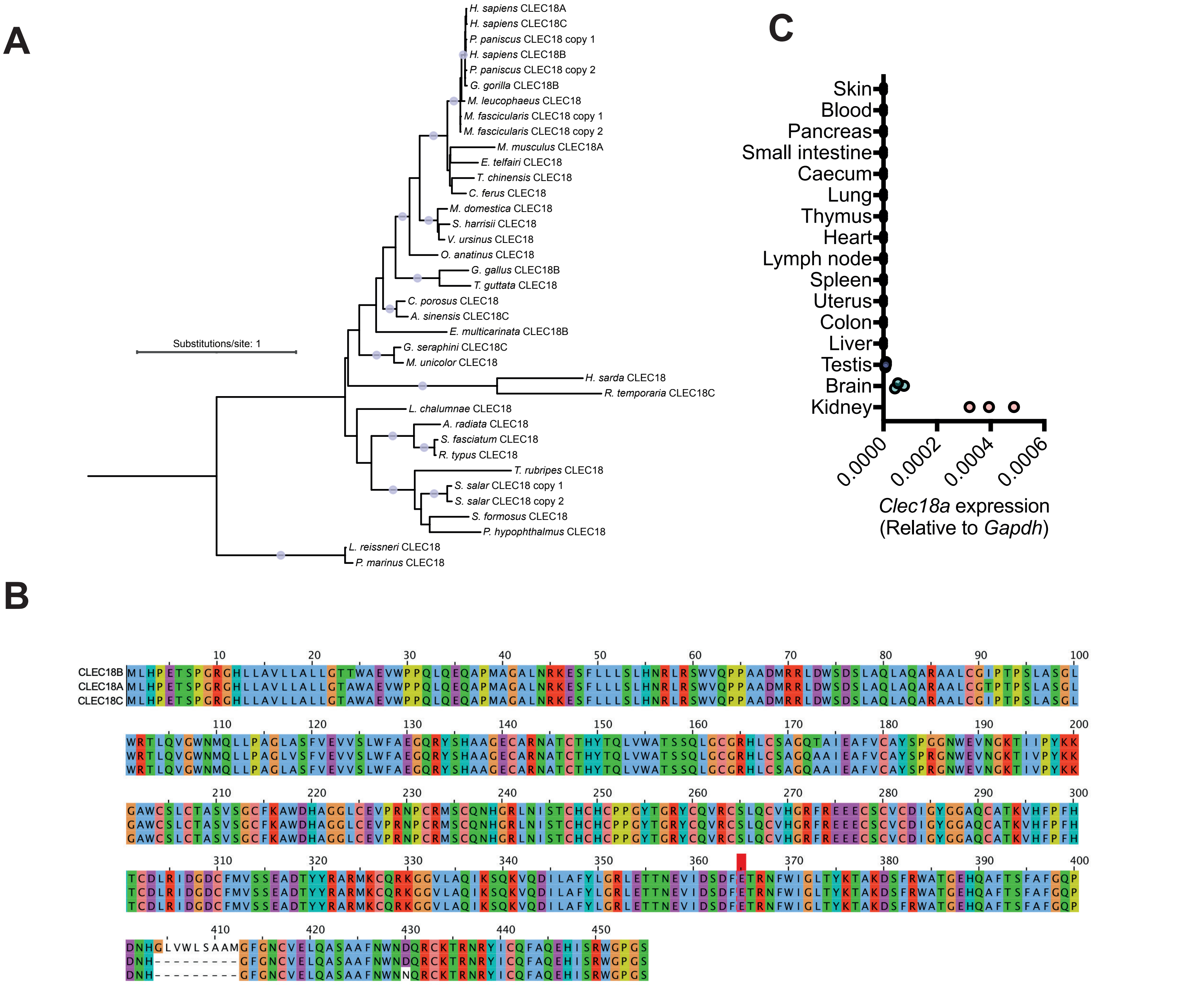

Supplementary Figure 1. Conservation and expression of CLEC18A

A. Maximum Likelihood phylogenetic tree based on a multiple sequence alignment of selected CLEC18 protein sequences. Branches that are supported by SH-aLRT ≥ 80% and UFboot2 ≥ 95% are indicated by a grey dot.

B. Multiple sequence alignment of human CLEC18A, CLEC18B and CLEC18C proteins colored according to the Clustal scheme.

C. *Clec18a* expression in mouse organs determined by RT-qPCR. Organs were always harvested from there individual mice.

Supplementary Figure 2.

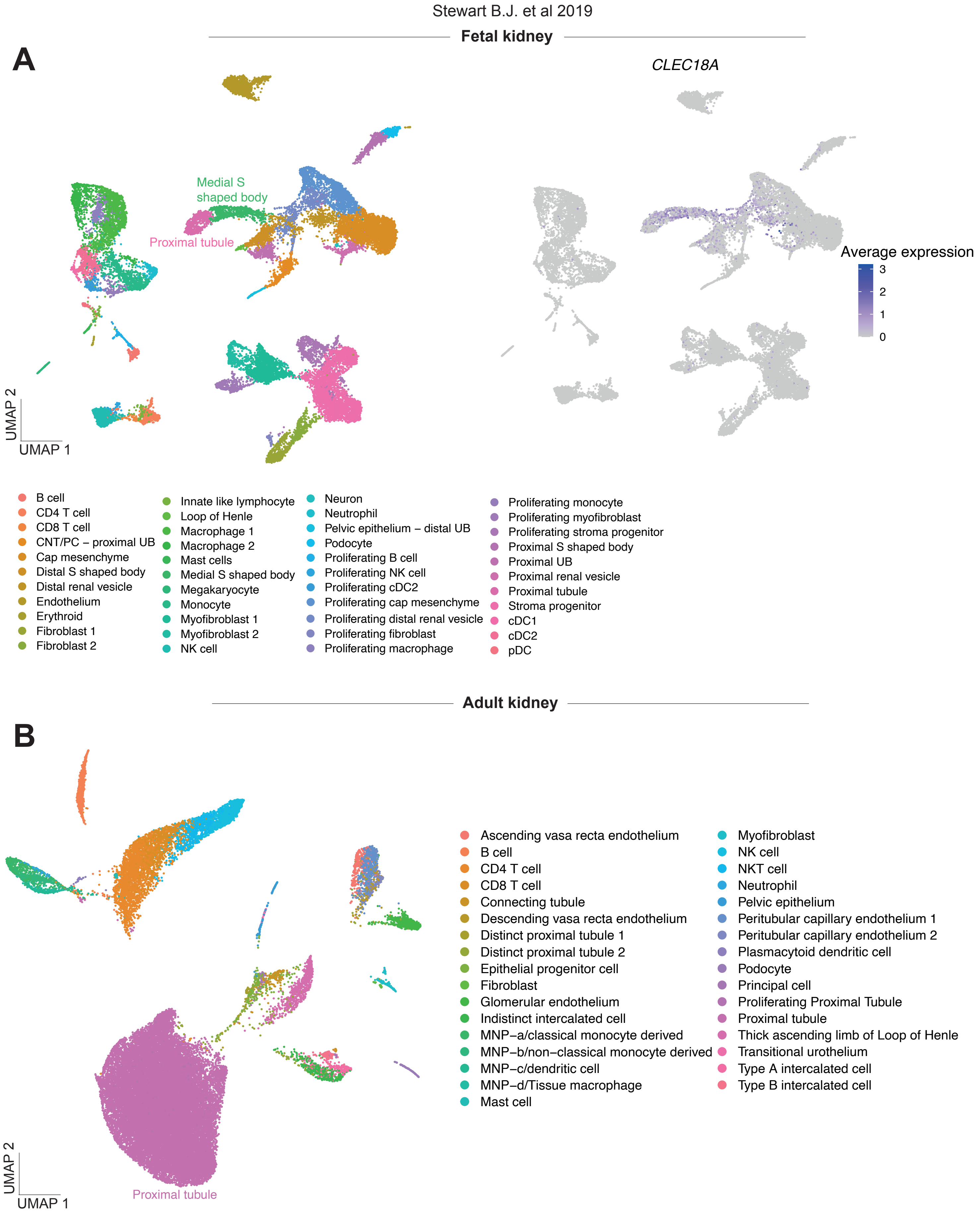

Supplementary Figure 2. scRNA-seq clustering of fetal kidney and adult kidney cells as accoridng to Stewart B.J. et al 2019 [20].

A. Re-analysis of scRNA-seq data from the kidney cell atlas [18] showing clustering of fetal kidney cells and *CLEC18A* expression in the full fetal kidney. Cell populations of interest, proximal tubule cells and medial S shaped body cells, are highlighted on the UMAP. Abbreviations: UMAP = Unifold manifold approximation and projection, CNT = Connecting tubule, PC = Principal cell, UB = Ureteric bud, NK cell = Natural killer cell, cDC1 = Conventional dendritic cell type 1, cDC2 = Conventional dendritic cell type 2, pDC = Plasmacytoid dendritic cell.

B. Re-analysis of scRNA-seq data from the kidney cell atlas [18] showing clustering of adult kidney cells. Cell populations of interest, proximal tubule cells, are highlighted on the UMAP. Abbreviations: UMAP = Unifold manifold approximation and projection, MNP = Mononuclear phagocyte, NK cell = Natural killer cell.

Supplementary Figure 3.

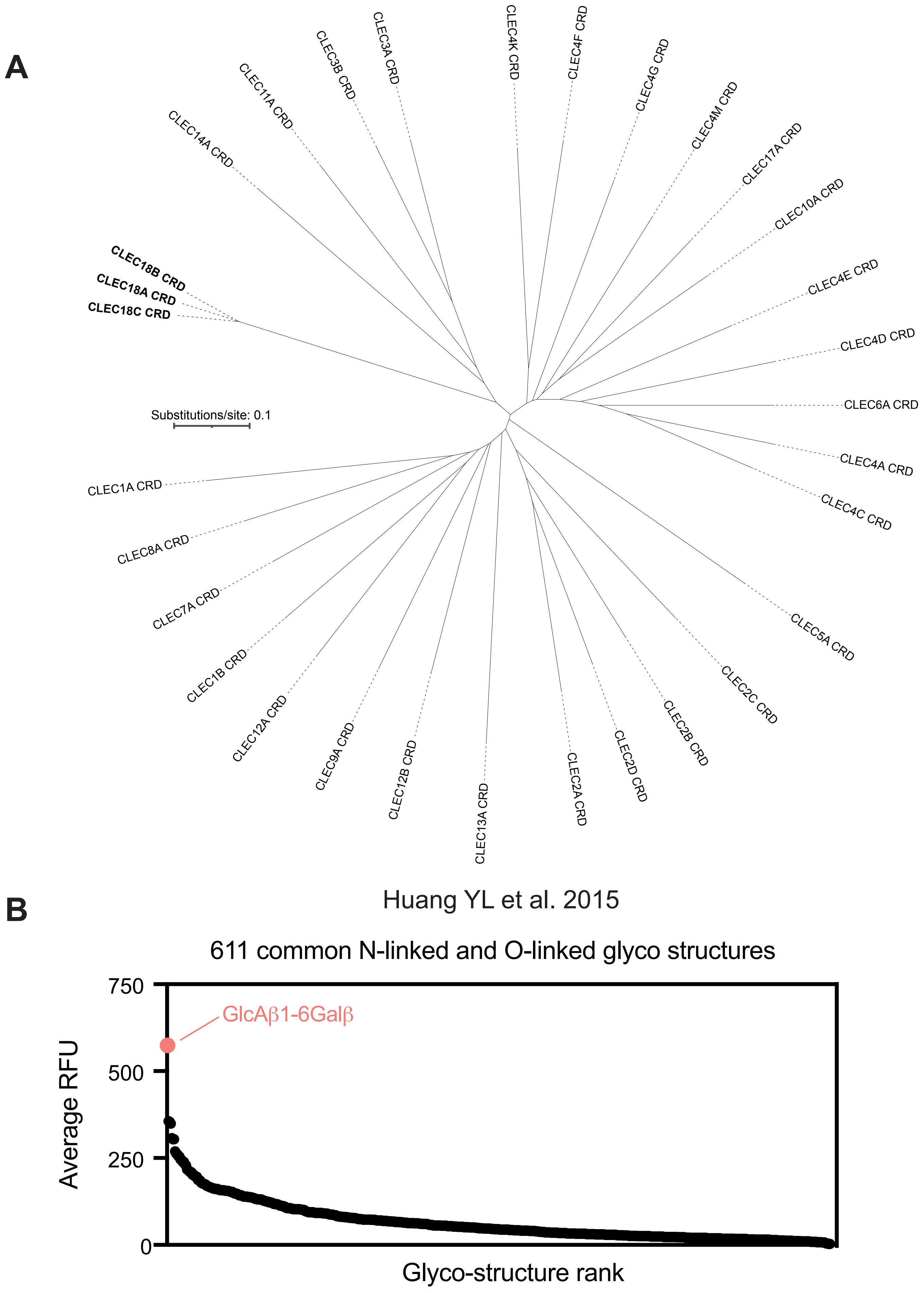

Supplementary Figure 3. CLEC18 CRDs have unique phylogeny and ligands

A. Unrooted neighbourhood joining phylogenetic tree based on the multiple sequence alignment of all included C-type lectin CRD sequences. Abbreviations: CRD = Carbohydrate recognition domain.

B. Re-analysis of a recombinant CLEC18A used on a GlycoArray containing 611 common N-linked and O-linked glyco structures [13].

Supplementary Figure 4.

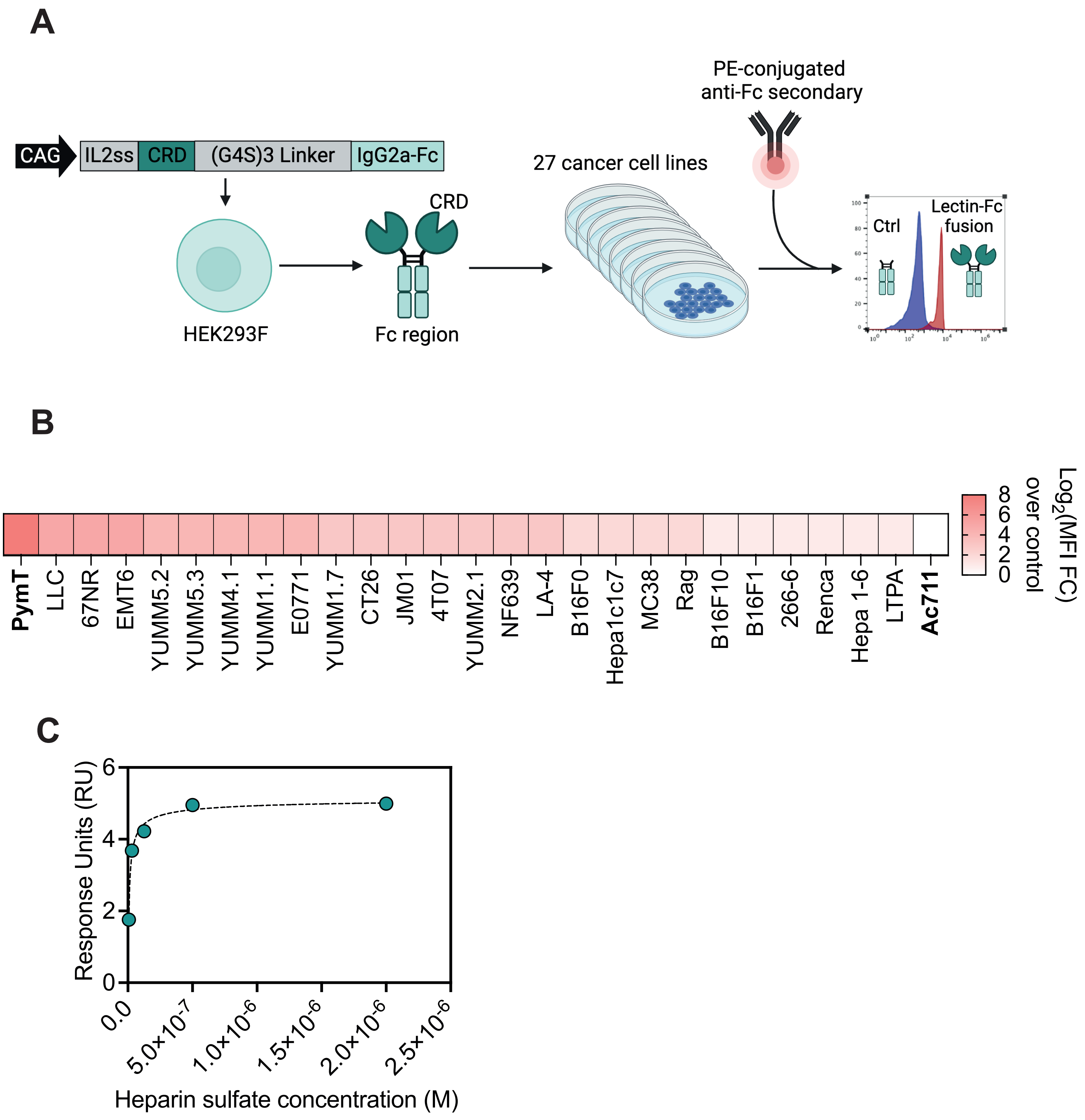

Supplementary Figure 4. CLEC18A-Fc binds cell lines with varying efficiency

A. Schematic of vector used for CLEC18A-Fc expression, subsequent expression and purification, and flow cytometry based readout used to identify cell lines CLEC18A-Fc binds to. Schematic created with BioRender.com.

B. Binding affinity of CLEC18A-Fc against a selection of cancer cell lines. The data represents the average of three independent experimental replicates.

C. Kinetic dose-response curve for the different response units obtained from varying heparin sulfate concentrations in surface plasmon resonance measurements

Supplementary Figure 5.

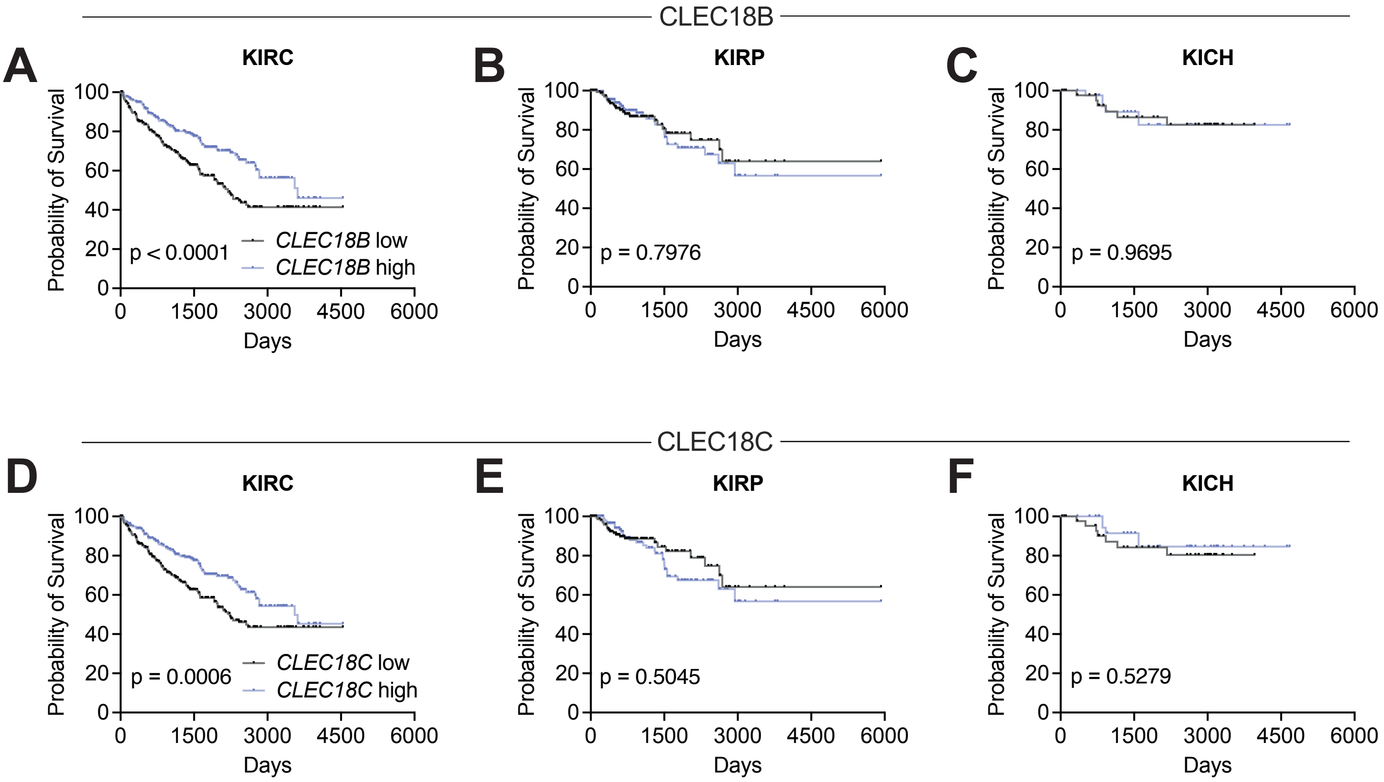

Supplementary Figure 5. CLEC18 exclusively promotes survival in ccRCC/KIRC

A. Kaplan meier survival curve for high vs low *CLEC18B* expression (high and low expression attributed based on the median) in KIRC. P value calculated with a Log rank test. Abbreviations: KIRC = Clear cell renal cell carcinoma.

B. Kaplan meier survival curve for high vs low *CLEC18B* expression (high and low expression attributed based on the median) in KIRP. P value calculated with a Log rank test. Abbreviations: KIRP = Papillary renal cell carcinoma.

C. Kaplan meier survival curve for high vs low *CLEC18B* expression (high and low expression attributed based on the median) in KICH. P value calculated with a Log rank test. Abbreviations: KICH = Chromophobe renal cell carcinoma.

D. Kaplan meier survival curve for high vs low *CLEC18C* expression (high and low expression attributed based on the median) in KIRC. P value calculated with a Log rank test. Abbreviations: KIRC = Clear cell renal cell carcinoma.

E. Kaplan meier survival curve for high vs low *CLEC18C* expression (high and low expression attributed based on the median) in KIRP. P value calculated with a Log rank test. Abbreviations: KIRP = Papillary renal cell carcinoma.

F. Kaplan meier survival curve for high vs low *CLEC18C* expression (high and low expression attributed based on the median) in KICH. P value calculated with a Log rank test. Abbreviations: KICH = Chromophobe renal cell carcinoma.

Supplementary Figure 6.

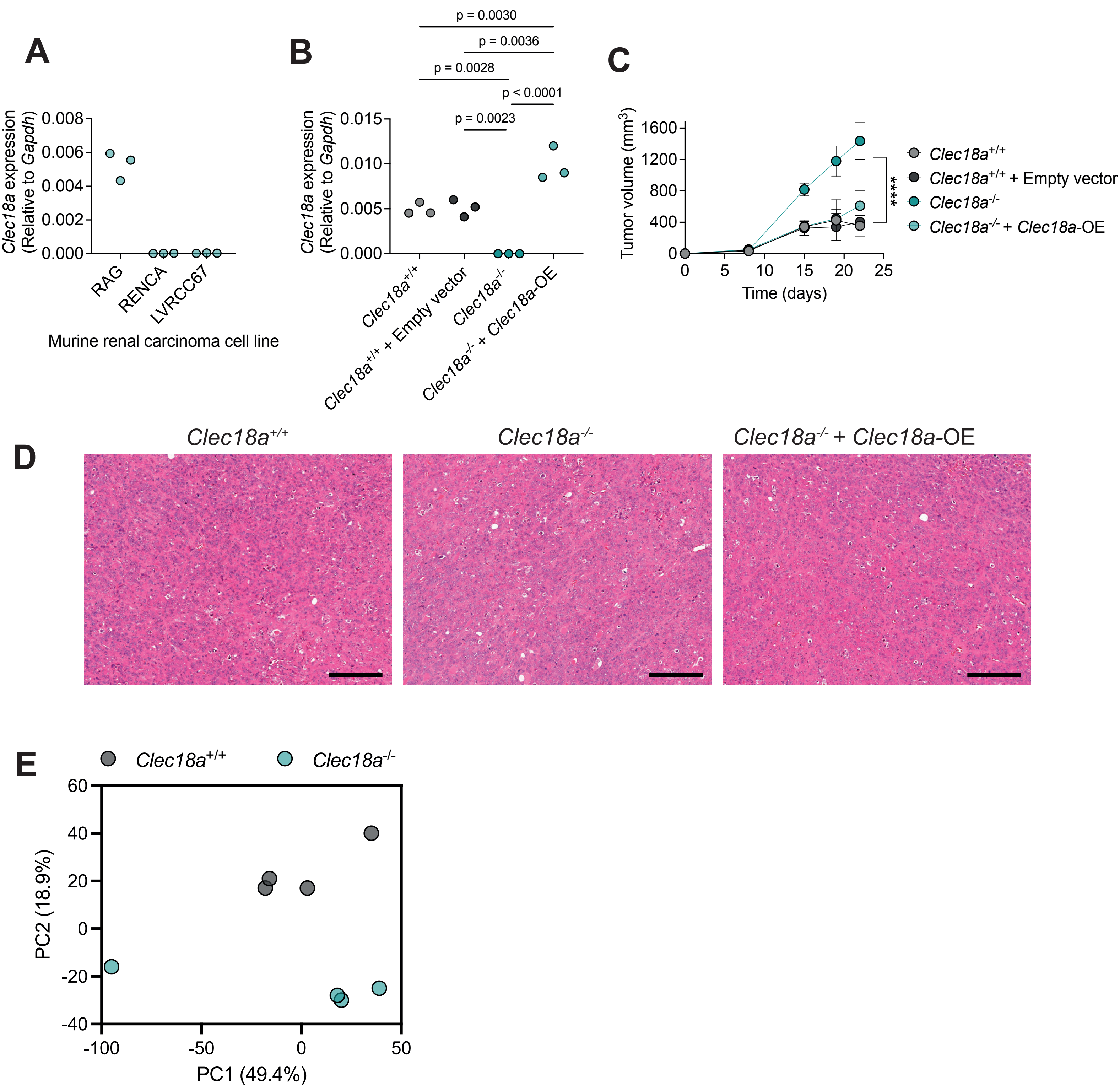

Supplementary Figure 6. CLEC18A expression restricts tumor progression in a murine model of renal carcinoma

A. *Clec18a* expression values in various murine renal cell cacinoma cell lines assessed with RT-qPCR. RAG and RENCA cell lines were obtained from ATCC and LVRCC67 was obtained from Rappold, P., L. Vuong et al 2022 [68].

B. Assessment of *Clec18a* expression levels in wild-type *Clec18a*$^{+/+}$ RAG cells transfected with an empty control vector and *Clec18a*$^{-/-}$ RAG cells rescued with an overexpression vector (*Clec18a*$^{-/-}$ + *Clec18a*-OE). P values were calculated using one-way ANOVA with Tukey's multiple comparisons test

C. Tumor growth curve kinetics of *Clec18a*$^{+/+}$ (n = 4), *Clec18a*$^{+/+}$ + Empty vector (n = 5), *Clec18a*$^{-/-}$ (n = 4) and *Clec18a*$^{-/-}$ + *Clec18a*-OE (n = 5) renal adenocarcinoma cell lines (RAG) in *Rag2*$^{-/-}$ *Il2rg*$^{-/-}$ mice. P values were calculated using two-way ANOVA with Šídák's multiple comparisons test. Error bars represent S.E.M.

D, Representative H&E staining of subcutaneous *Clec18a*$^{+/+}$, *Clec18a*$^{-/-}$ and *Clec18a*$^{-/-}$ + *Clec18a*-OE renal adenocarcinomas from *Rag2*$^{-/-}$ *Il2rg*$^{-/-}$ mice. Scale bars = 100 µm.

E. Principal component analysis of RNA sequencing samples. Abbreviations: PC = Principal component.
